# Mechanisms of Pancreatic Injury Induced by Basic Amino Acids Differ Between L-Arginine, L-Ornithine, and L-Histidine

**DOI:** 10.3389/fphys.2018.01922

**Published:** 2019-01-15

**Authors:** Xiaoying Zhang, Tao Jin, Na Shi, Linbo Yao, Xinmin Yang, Chenxia Han, Li Wen, Dan Du, Peter Szatmary, Rajarshi Mukherjee, Tingting Liu, Qing Xia, David N. Criddle, Wei Huang, Michael Chvanov, Robert Sutton

**Affiliations:** ^1^Department of Integrated Traditional Chinese and Western Medicine, Sichuan Provincial Pancreatitis Centre and West China-Liverpool Biomedical Research Centre, West China Hospital, Sichuan University, Chengdu, China; ^2^Liverpool Pancreatitis Study Group, Royal Liverpool University Hospital, Institute of Translational Medicine, University of Liverpool, Liverpool, United Kingdom; ^3^West China-Washington Mitochondria and Metabolism Centre, West China Hospital of Sichuan University, Chengdu, China; ^4^Department of Cellular and Molecular Physiology, University of Liverpool, Liverpool, United Kingdom

**Keywords:** acute pancreatitis, amino acids, mitochondria, caffeine, cyclophilin D, necroptosis, G-protein coupled receptors class C

## Abstract

Pancreatic acinar cells require high rates of amino acid uptake for digestive enzyme synthesis, but excessive concentrations can trigger acute pancreatitis (AP) by mechanisms that are not well understood. We have used three basic natural amino acids L-arginine, L-ornithine, and L-histidine to determine mechanisms of amino acid-induced pancreatic injury and whether these are common to all three amino acids. Caffeine markedly inhibited necrotic cell death pathway activation in isolated pancreatic acinar cells induced by L-arginine, but not L-ornithine, whereas caffeine accelerated L-histidine-induced cell death. Both necroptosis inhibitors of RIPK1 and RIPK3 and a necroptosis activator/apoptosis inhibitor z-VAD increased cell death caused by L-histidine, but not L-arginine or L-ornithine. Cyclophilin D knock-out (Ppif^-/-^) significantly attenuated cell death induced by L-histidine, but not L-arginine, or L-ornithine. Allosteric modulators of calcium-sensing receptor (CaSR) and G-protein coupled receptor class C group 6 member A (GPRC6A) had inhibitory effects on cell death induced by L-arginine but not L-ornithine or L-histidine. We developed a novel amino acid-induced AP murine model with high doses of L-histidine and confirmed AP severity was significantly reduced in Ppif^-/-^ vs. wild type mice. In L-arginine-induced AP neither Ppif^-/-^, caffeine, or allosteric modulators of CaSR or GPRC6A reduced pancreatic damage, even though CaSR inhibition with NPS-2143 significantly reduced pancreatic and systemic injury in caerulein-induced AP. These findings demonstrate marked differences in the mechanisms of pancreatic injury induced by different basic amino acids and suggest the lack of effect of treatments on L-arginine-induced AP may be due to conversion to L-ornithine in the urea cycle.

## Introduction

Pancreatic acinar cells that generate pancreatic enzymes display the fastest protein synthesis among normal cell types (Leblond et al., 1957). To meet this need the exocrine pancreas accumulates amino acids (AAs) to a greater extent than most other tissues (Longnecker, 1977). This affinity to AAs may have application in the development of pancreas-specific drugs. At the same time, pancreatic toxicity has been observed in animals administered some unnatural AAs or large amounts of several natural AAs. High doses of basic AAs (most often L-arginine) are widely used in animal models of acute pancreatitis (AP), although the mechanisms of the pancreatic damage caused by AAs are not well understood. It has been reported that calcium independent mitochondrial injury precedes other early events in the induction of AP by L-lysine, L-ornithine, and L-arginine (Biczo et al., 2011b, 2018; Kui et al., 2014). However, in some reports the ultrastructural changes in response to toxic doses of AAs were detected earlier in the endoplasmic reticulum (ER) than mitochondrial abnormalities (Kishino and Kawamura, 1984; Kubisch et al., 2006). In a range of experimental models of AP, including caerulein hyperstimulation, ductal infusion of bile acid, or fatty acid plus ethanol, it has been firmly established that sustained cytosolic calcium elevation initiates deleterious events in cells (Ward et al., 1996; Perides et al., 2010; Huang et al., 2014; Wen et al., 2015). Caffeine, which in pancreatic acinar cells blocks calcium signaling mediated by IP_3_ receptors, has been found to be a strong protector in these non-AA models of AP (Huang et al., 2017). Downstream of cytosolic calcium overload, inhibition of the mitochondrial permeability transition pore (MPTP) by genetic deletion or pharmacological inhibition of peptidyl prolyl isomerase D (cyclophilin D, encoded by *Ppif* gene) prevents ATP loss and improves biochemical, immunological and histopathological parameters in non-AA models of AP (Shalbueva et al., 2013; Mukherjee et al., 2016). Recently Ppif^-/-^ has been shown to reduce the severity of AP induced by L-arginine through reduction of ATP synthesis not dependent on calcium overload (Biczo et al., 2018). The effect of Ppif^-/-^, however, has not been tested in acinar cells and mice challenged by other AAs.

L-AAs serve as ligands for family C (or class 3) G-protein coupled receptors (GPCR), including CaSR (calcium sensing receptor), and GPRC6A (G protein-coupled receptor family C group 6 member A) (Conigrave and Hampson, 2006; Wellendorph et al., 2009). These two receptors have been reported to be expressed in the pancreas (Bruce et al., 1999; Racz et al., 2002; Wellendorph and Brauner-Osborne, 2004); CaSR mutations have been linked to chronic pancreatitis (Muddana et al., 2008). In other tissues these receptors respond to tens and hundreds of micromolar concentrations of AAs, with CaSR activated primarily by aromatic AAs such as phenylalanine, tryptophan, and histidine while GPRC6A more sensitive to arginine and lysine (Conigrave and Hampson, 2006; Wellendorph et al., 2009). In the experimental conditions of AP the concentration of AA exceeds 10 mM. The role of CaSR and GPRC6a, however, has never been assessed in relation to AA-induced pancreatic damage.

Since it has been noted that conversion of L-arginine to L-ornithine is an important step in the induction of AP by L-arginine (ARG-AP) (Biczo et al., 2010), we have compared the effects of caffeine, modulators of necroptosis, cyclophilin D knock-out, and allosteric modulators of GPCR class C (CaSR and GPRC6A) on pancreatic acinar cell death and AP induced by three basic AAs L-arginine, L-ornithine, and L-histidine, including on a new model of L-histidine-induced AP (HIS-AP).

## Materials and Methods

### Ethics Statement

All animal studies were ethically reviewed and approved according to Ethics Committee of West China Hospital of Sichuan University (2017065A) or Local Animal Welfare Committee at University of Liverpool which followed United Kingdom Animals (Scientific Procedures) Act 1986 and approved by United Kingdom Home Office (PPL 40/3320, renewed as 70/8109). Human pancreatic samples were obtained with written informed consent as approved by Liverpool Adult Local Research Ethics Committee (Ref: 03/12/242/A).

### Animals and Reagents

Male C57BL/6J mice (25–30 g) were from Huafukang Bioscience Co., Ltd. (Beijing, China) or Charles River UK Ltd. (Oxford, United Kingdom). Cyclophilin D-deficient mice were generated by targeted disruption of the *Ppif* gene and generously provided by Dr. D. Yellon (University College London, United Kingdom) and Dr. M. A. Forte (Oregon Health and Sciences University, United States). Genotyping of the mice was confirmed as described (Armstrong et al., 2018). Animals were maintained at 22 ± 2°C and exposed to a 12 h light-dark cycle, fed with standard laboratory chow and water, allowed to acclimatize for a minimum of 1 week. For *in vivo* experiments 10 week old wild type (Wt) or Ppif^-/-^ mice (>25 g) were used.

Propidium iodide (PI) was from Molecular Probes (OR, United States); Boc-Gln-Ala-Arg-MCA was from Enzo Life Sciences (NY, United States); protease inhibitors were from Roche GmbH (Mannheim, Germany); interleukin (IL)-6 Quantikine ELISA Kit from R&D Systems (Abingdon, United Kingdom); modulators of necroptosis (necrostatin-1, GSK-872, z-VAD, necrosulfonamide [NSA]) were from MedChemExpress (NJ, United States); NPS-2143 and R-568 were from Tocris (Bristol, United Kingdom); 2-oxo-2-(2-phenyl-1H-indol-3-yl) ethyl-3-aminopyrazine-2-carboxylate (Cpd1) was from Enamine (Kiev, Ukraine); rabbit polyclonal antibodies against CaSR and GPRC6A were from Abcam (ab18200; Cambridge, United Kingdom) and Acris (AP06864-PUN, Upper Heyford, United Kingdom), respectively; secondary Alexa Fluor^®^ 488 goat anti-rabbit was from Molecular Probes (A11034, OR, United States); ALZET^®^ osmotic mini-pump was from Charles River UK Ltd. (model 1003D; Oxford, United Kingdom). If not otherwise indicated, other reagents were at highest grade from Sigma (Dorset, United Kingdom).

### Preparation of Isolated Pancreatic Acinar Cells

Murine pancreatic acinar cells were isolated by digestion with purified collagenase (200 U/ml, 20 min at 37°C) and pipetting, as described (Chvanov et al., 2018). They were kept in extracellular solution (140 mM NaCl, 4.7 mM KCl, 1.13 mM MgCl2, 1.2 mM CaCl_2_, 10 mM 4-[2-hydroxyethyl]-1-piperazineethanesulfonic acid [HEPES], and 10 mM D-glucose at pH 7.35–7.45). For the AA experiments, in order to maintain the osmolarity, the concentration of NaCl was reduced (to 120 mM for L-arginine and L-ornithine hydrochlorides and to 130 mM for L-histidine free base). Human pancreatic acinar cells were isolated by digestion of human pancreatic samples cut into ∼1 cm × 1 cm × 1 cm pieces, using the same collagenase in several digestion rounds: 20 min followed by 20 and then another 30 min. All cells pipetted out after each digestion step were pooled together and put in the extracellular solution on ice.

### Immunofluorescence

Cells were plated on 35 mm glass-bottomed dishes (MatTek Corporation, Ashland, MA, United States) fixed in 4% paraformaldehyde for 30 min, permeabilised with 0.1% Triton X-100 for 10 min, and non-specific binding was blocked with 10% goat serum and 1% bovine serum albumin in phosphate-buffered saline for 1 h at room temperature. Cells were incubated with monoclonal primary antibodies at 1:100 dilution. Nuclei were stained with Hoechst-33342 (5 μg/ml) for 10 min. After staining with secondary antibody for 20 min (at 1:1000), the cells were imaged on TCS-SP2 confocal microscope (Leica Microsystems, Germany) with 1÷2 x airy unit pinhole.

### Necrotic Cell Death Assay

Necrotic cell death pathway activation in pancreatic acinar cells was reflected by the intensity of fluorescent dye PI (10 μg/ml final concentration) uptake by the nuclei of necrotic/dying cells measured on POLARstar Omega Plate Reader (BMG Labtech, Germany) at 37°C using 96 flat bottom wells as previously described (Huang et al., 2017). All fluorescence measurements are expressed as fold changes from the time zero fluorescence (*F/F_0_* ratio). The time to half-maximum response for each experiment in each treatment group was calculated and then statistically analyzed to get the information on the speed of process rather than amplitude (see Supplementary Figure S1A). The concentration of all AAs used was 20 mM, the concentration of DMSO in all solutions was 0.05%.

### Experimental AP Models

Mice received: (1) two intraperitoneal injections of L-arginine monohydrochloride (8%, 4 g/kg) at the room temperature at 1 h interval (ARG-AP) as described (Dawra et al., 2007; Kui et al., 2015); (2) two intraperitoneal injections L-histidine free base (7%, 4 g/kg) freshly prepared by microwaving and cooling to physiological temperature immediately prior to the injection at 1 h interval (HIS-AP); (3) two intraperitoneal injections L-ornithine (8%, 4 g/kg); or (4) seven intraperitoneal injections of caerulein (10 μg/ml, 50 μg/kg) at hourly intervals (CER-AP).

Control mice for each model received saline injections under the same condition with the same regimen, respectively. For the ARG-AP and its controls, mice were humanly sacrificed at 72 h after the first L-arginine/saline injection; for HIS-AP and it controls, mice were sacrificed at various time points to understand the histopathological patterns of this model; CER-AP and its controls were sacrificed at 12 h after the first caerulein/saline injection.

### Pharmacological Interventions

In the treatment groups of ARG-AP, NPS-2143, and calindol were given intraperitoneally at 1.1 mg/kg 2 h prior to the first L-arginine injection and every 12 h after that (0.9 μl DMSO per 30 g mouse with each injection); Cpd1 (0.5 M stock in DMSO:PEG200 [1:1]) was administered by using subcutaneously implanted, filled and primed osmotic mini-pump (the delivery rate of Cpd1 was 0.5 μmol/h, with 0.5 μl/h DMSO and PEG released into circulation) according to the manufacturer’s instruction; seven intraperitoneal injections of caffeine (25 mg/kg) at hourly interval begun 24 and 48 h after the first L-arginine injection. For CER-AP, NPS-2143 was also given intraperitoneally at 1.1 mg/kg with the first caerulein injection.

### Histopathology and Immunohistochemistry

Pancreatic tissue was fixed in 10% formalin, embedded in paraffin and stained (H&E). Pancreatic histopathological score was performed on 10 random fields (magnification ×200) by two independent investigators who were unaware of the study design, grading 0–4 for oedema, inflammatory cell infiltration and acinar necrosis, respectively (Ou et al., 2015). The overall pancreatic histopathology score was the sum of the individual scores. Primary antibodies were used at 1:50 and the secondary antibody at 1:1000 dilution. Images of cells and organelles were obtained on TCS-SP2 confocal microscope using a 488 nm laser line; the emission was recorded at wavelengths 505–530 nm.

### Enzyme Activity and IL-6 Measurement

Pancreatic trypsin activity was measured as described in homogenized tissue (Boc-Gln-Ala-Arg-MCA substrate; excitation 380 nm, emission 440 nm) (Nathan et al., 2005). Myeloperoxidase activity was determined as described (Dawra et al., 2008). Serum amylase and IL-6 were determined kinetically using a Roche automated clinical chemistry analyzer (GMI, Leeds, United Kingdom) and ELISA, respectively.

### Statistical Analysis

Data are presented as mean ± SEM in traces and vertical bars. Comparisons were made using two-tailed Student’s *t*-test: paired for caffeine and GPCR modulators *in vitro* (which were always run in parallel to non-treatment group) and unpaired for Ppif^-/-^ vs. Wt and necroptosis comparison *in vitro* and all *in vivo* data. Comparisons of between any two time points of the HIS-AP model were made using Mann–Whitney *U*-test. Statistical significance was set at *p* < 0.05.

## Results

### Caffeine Protects Against L-Arginine-Induced Acinar Cell Death *in vitro* but Not *in vivo*

**FIGURE 1:**
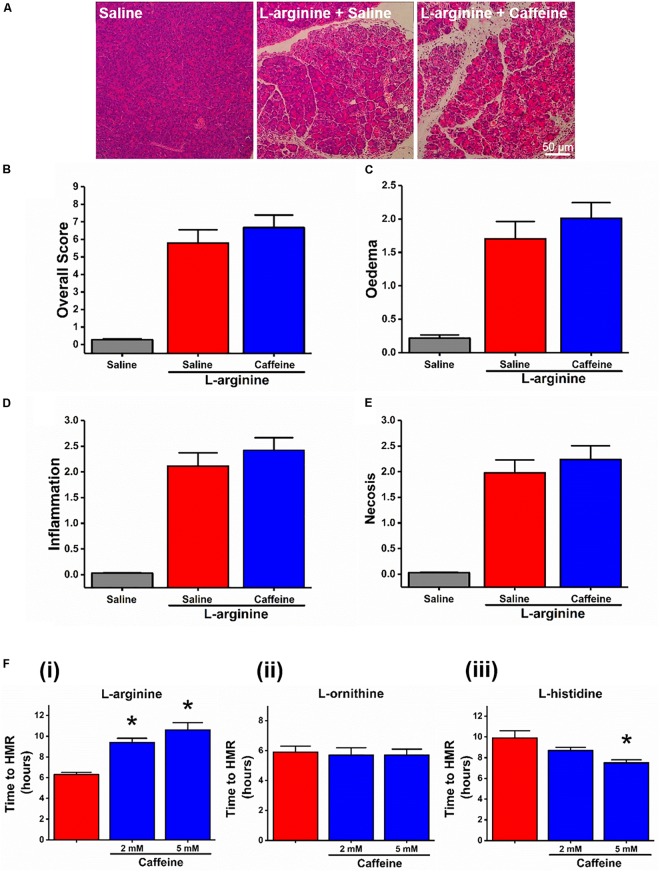
Effect of caffeine on L-arginine-induced acute pancreatitis *in vivo* and on cell death caused by basic amino acids *in vitro*. Mice received intraperitoneal injections of L-arginine (pH 7.4, 2 × 4 g/kg, at 1 h interval) with or without concomitant caffeine administration (7 × 25 mg/kg, at hourly intervals) begun at 24 and 48 h after the first L-arginine injection and sacrificed at 72 h: **(A)** representative images of pancreatic histopathology changes (H&E, ×200, scale bar 50 μm) and scores – **(B)** overall, **(C)** oedema, **(D)** inflammation, and **(E)** necrosis. **(F)** Effect of caffeine (2 and 5 mM) on necrotic cell death pathway activation (presented as time to half-maximal response [HMR] of propidium iodide [PI] uptake) of freshly isolated mouse pancreatic acinar cells caused by basic amino acids (all at 20 mM): **(i)** L-arginine, **(ii)** L-ornithine, and **(iii)** L-histidine. ^∗^*p* < 0.05 vs. L-arginine or L-histidine treatment only. Values are means ± SEM from ≥6 experiments/group (*in vitro*) or mice/group (*in vivo*).

We started with assessing the effect of caffeine administration on the severity of ARG-AP. In contrast to CER-AP, bile acid and fatty acid plus ethanol models (Huang et al., 2017), in ARG-AP caffeine had no significant effect for all the histopathological parameters assessed (all *p* > 0.05; Figures 1A–E). Next, we investigated the necrotic cell death pathway activation (evaluated by PI uptake) of pancreatic acinar cells in response to a range of AAs (all at 20 mM) *in vitro*. Caffeine (2 and 5 mM) dose-dependently inhibited (delayed) cell death elicited by L-arginine (both *p* < 0.01; Figure 1Fi). We used time to half maximal response as a representative parameter of the rate of cell death (see Supplementary Figure S1 for details of the calculation procedure and how it correlates with the full kinetic traces). Surprisingly, cell death responses to basic AAs L-ornithine and L-histidine were remarkably different in terms of sensitivity to caffeine. Caffeine (2 and 5 mM) did not significantly affected cell death caused by L-ornithine (both *p* > 0.05; Figure 1Fii) and accelerated cell death induced by L-histidine (*p* = 0.07 for 2 mM caffeine; *p* < 0.01 for 5 mM caffeine, Figure 1Fiii).

In addition, the modulators of necroptosis (RIP family members mediated programmed form of necrosis) did not alter the time course of necrotic cell death pathway activation caused by L-arginine and L-ornithine (Supplementary Figures S2A,B). However, several blockers of necroptosis (RIPK1 inhibitor necrostatin-1 at 50 μM and RIPK3 inhibitor GSK-872 at 3 μM) and a necroptosis activator/apoptosis inhibitor z-VAD (at 25 μM) increased the rate of necrosis caused by L-histidine (Supplementary Figure S2C; *p* < 0.01 for these compounds, *p* = 0.16 for MLKL and necroptosis inhibitor NSA, 0.5 μM).

### Cyclophilin D Knock-Out Does Not Protects Against L-Arginine-Induced Damage *in vitro* or *in vivo*

**FIGURE 2:**
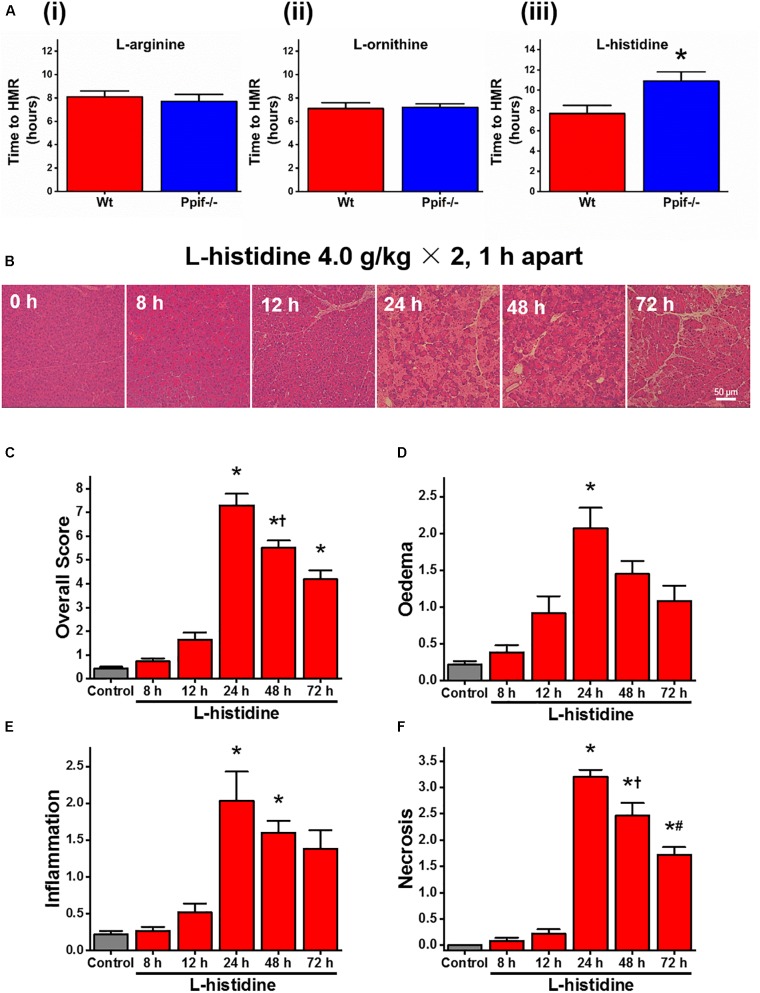
Effect of cyclophilin D knock-out on cell death caused by basic amino acids *in vitro* and L-histidine-induced acute pancreatitis *in vivo*. **(A)** Effect of cyclophilin D knock-out (Ppif^-/-^) in comparison with wild type (Wt) on necrotic cell death pathway activation (presented as time to half-maximal response [HMR] of propidium iodide [PI] uptake) of freshly isolated mouse pancreatic acinar cells caused by basic amino acids (all at 20 mM): **(i)** L-arginine, **(ii)** L-ornithine, and **(iii)** L-histidine (^∗^*p* < 0.05 vs. L-histidine treatment only). Mice received intraperitoneal injections of 7% L-histidine (pH 7.4, 2 × 4 g/kg, at 1 h intervals) and were sacrificed at indicated time points after the first L-histidine injection: **(B)** representative images of pancreatic histopathology changes (H&E, ×200, scale bar 50 μm) and scores – **(C)** overall, **(D)** oedema, **(E)** inflammation, and **(F)** necrosis (^∗^*p* < 0.05 vs. saline control, L-histidine 8 h or L-histidine 12 h, ^†^*p* < 0.05 vs. L-histidine 24 h, ^#^*p* < 0.05 vs. L-histidine 48 h). Values are means ± SEM from 3–6 experiments/group (*in vitro*) or mice/group (*in vivo*).

Given the difference between the effect of caffeine on L-arginine induced responses *in vitro* and *in vivo*, we next decided to test the effect of cyclophilin D knock-out in both settings. We found no major differences in the rate of cell death in the presence of 20 mM L-arginine or L-ornithine between freshly isolated pancreatic acinar cells from Ppif^-/-^ and the matched Wt mice (both *p* > 0.05; Figures 2Ai,2Aii). However, the cells challenged with L-histidine from Ppif^-/-^ mice had significantly delayed necrosis compared to Wt mice (*p* < 0.05; Figure 2Aiii).

All mice were dead within few hours after L-ornithine injections. In order to directly compare the effect of cyclophilin D knock-out on L-arginine and L-histidine *in vivo*, we developed a novel AA-induced AP model by L-histidine, HIS-AP (Figure 2B). In HIS-AP the damage to the pancreas, as assessed by histopathology scores, peaked at 24 h after the first L-histidine injection, showing a subsequent decrease at 48 and 72 h (Figures 2C–F). The peak of the acinar cell necrosis in the ARG-AP was reported as 72 h (Dawra et al., 2007), so we did all subsequent comparisons in both ARG-AP and HIS-AP at this time point.

**FIGURE 3:**
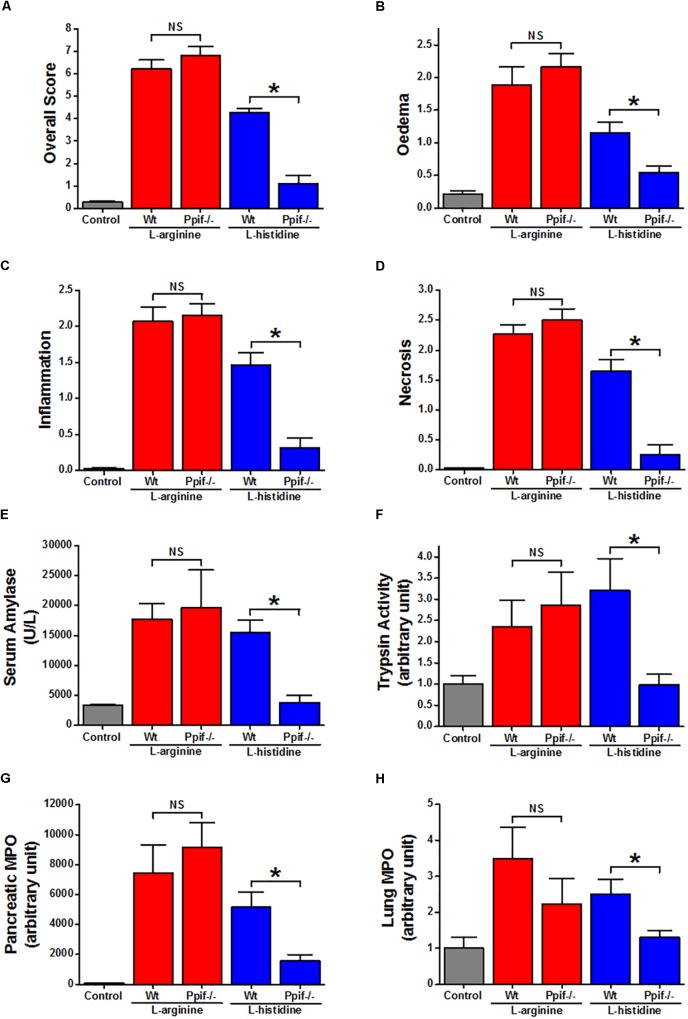
Comparison of the effect of cyclophilin D knock-out on acute pancreatitis caused by L-arginine and L-histidine. Cyclophilin D knock-out (Ppif^-/-^) or wild type (Wt) mice received either intraperitoneal injections of either 8% L-arginine or 7% L-histidine (both: pH 7.4, 2 × 4 g/kg, at 1 h intervals) and were sacrificed at 72 h after the first L-arginine/L-histidine injection: **(A)** overall, **(B)** oedema, **(C)** inflammation and **(D)** necrosis histopathological scores; **(E)** serum amylase, **(F)** pancreatic trypsin activity, **(G)** pancreatic myeloperoxidase (MPO) activity and **(H)** lung MPO activity. ^∗^*p* < 0.05 vs. L-histidine with Wt mice. Values are means ± SEM of 6–8 animals per group.

There were no significant differences in severity parameters in ARG-AP between Ppif^-/-^ and Wt mice (Figure 3, red vertical bars). Both groups had dramatic increase of overall histopathological score (Figure 3A) and its subclass scores: oedema (Figure 3B), inflammatory cell infiltration (Figure 3C) and acinar cell necrosis (Figure 3D) compared to the controls. In parallel, serum amylase (Figure 3E), trypsin activity (Figure 3F), pancreatic (Figure 3G) and lung (Figure 3H) myeloperoxidase activity were greatly increased, and their magnitudes were similar between the Ppif^-/-^ and Wt mice. Consistent increase of histopathological and biochemical severity markers were also observed in HIS-AP, albeit less severe when compared to the ARG-AP at this time point (Figure 3, blue vertical bars). In Ppif^-/-^ mice all severity parameters were greatly attenuated compared to the Wt mice for this model. The representative histopathological images for all groups are shown in Supplementary Figure S3.

### Varied Effects of Allosteric Modulators of CaSR and GPRC6A on the Cell Death Induced by Basic AAs *in vitro* and No Effect on ARG-AP

**FIGURE 4:**
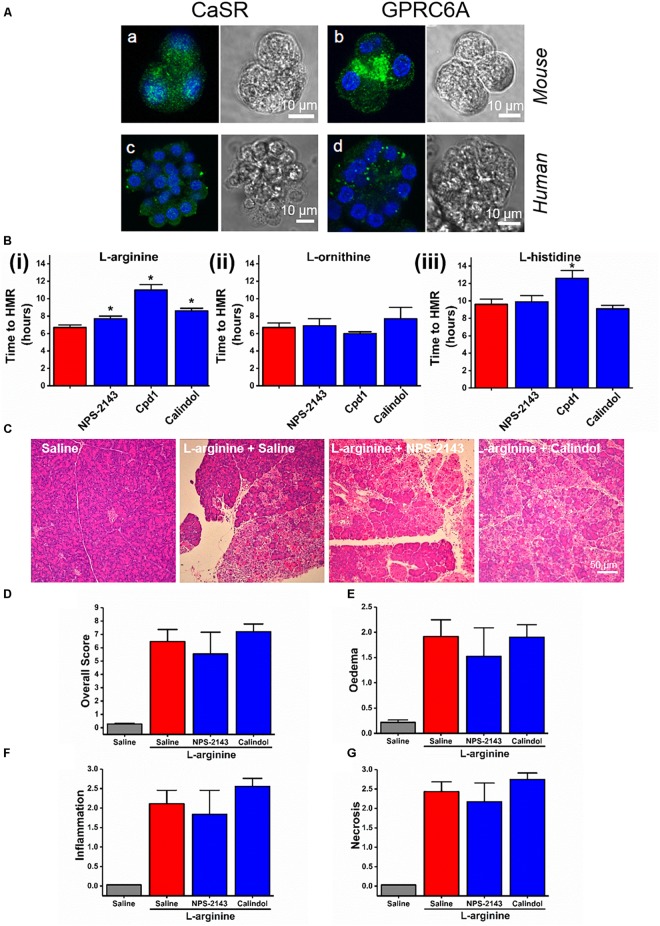
Effect of allosteric modulators of GPCR Class C on pancreatic acinar cell death caused by basic amino acids *in vitro* and on L-arginine-induced acute pancreatitis *in vivo*. **(A)** Images of immunohistochemistry staining against CaSR and GPRC6A in mouse and human fixed pancreatic acinar cells. **(B)** Effect of calcilytic NPS-2143 (1 μM), GPRC6A antagonist Cpd1 (50 μM), and calcimimetic calindol (1 μM) on necrotic cell death activation (presented as time to half-maximal response [HMR] of propidium iodide [PI] uptake) caused by basic amino acids (all at 20 mM): **(i)** L-arginine, **(ii)** L-ornithine, and **(iii)** L-histidine. Mice received intraperitoneal injections of either 8% L-arginine (pH 7.4, 2 × 4 g/kg, at 1 h interval) and were sacrificed at 72 h after the first L-arginine injection: **(C)** effects of calcilytic NPS-2143 and calcimimetic calindol *in vivo*, representative images of pancreatic histopathology changes (H&E, ×200, scale bar 50 μm) and scores – **(D)** overall, **(E)** oedema, **(F)** inflammation, and **(G)** necrosis. ^∗^*p* < 0.05 vs. L-arginine or L-histidine treatment only. Values are means ± SEM from ≥6 experiments/group (*in vitro*) or mice/group (*in vivo*).

In fixed dissociated cells, immunohistochemistry staining with relevant antibodies confirmed the expression of both CaSR and GPRC6A in both mouse and human pancreatic acinar cells (Figure 4A). Most of the anti-CaSR and anti-GPRC6A staining of the acinar cells permeabilised with Triton X-100 was intracellular in the granular/apical areas, coming presumably from the immature protein or the protein that is on its way to the plasma membrane.

We looked into the role of CaSR and GPRC6A in the basic AA (all 20 mM) toxicity in the pancreas, using small molecule allosteric modulators of these two receptors. NPS-2143, which is CaSR inhibitor (calcylitic) and weak GPRC6A antagonist, at 1 μM delayed cell death *in vitro* in the presence of L-arginine (*p* < 0.01; Figure 4Bi), but not but not L-ornithine (*p* > 0.05; Figure 4Bii) or L-histidine (*p* > 0.05; Figure 4Biii). CaSR activator (calcimimetic) calindol at 1 μM also delayed cell death caused by L-arginine (*p* < 0.001; Figure 4Bi). There was no protection by calindol against L-ornithine or L-histidine-induced cell death (Figures 4Bii,4Biii). Another calcimimetic R-568 at 1 μM showed exactly the same spectrum of effects on AA-induced responses as calindol (data not shown).

The GPRC6A antagonist Cpd1 (Johansson et al., 2015) at 50 μM substantially delayed cell death caused by L-arginine (*p* < 0.0001; Figure 4Bi) and by L-histidine (*p* < 0.001; Figure 4Biii), but not by L-ornithine (*p* > 0.05; Figure 4Bii).

**FIGURE 5:**
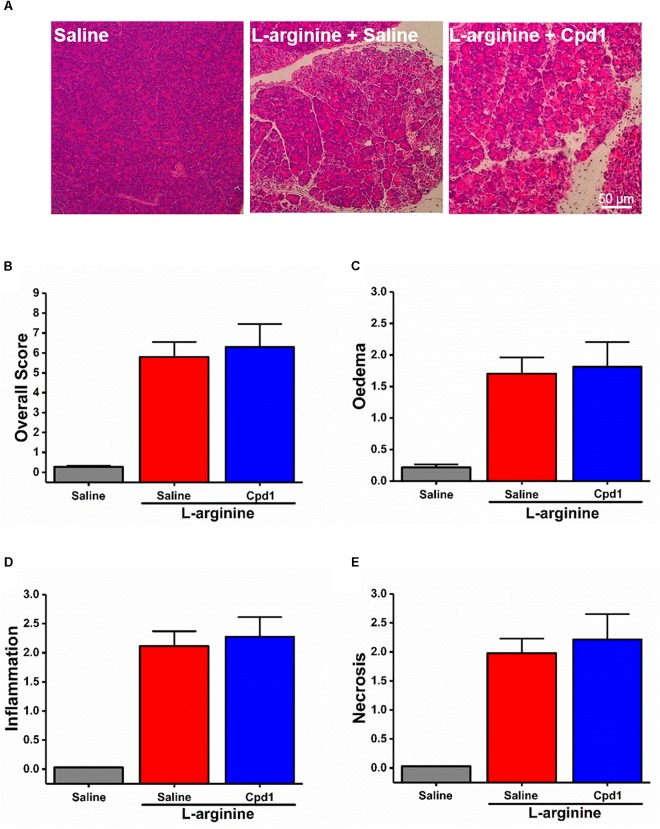
Effect of allosteric modulators of GPRC6A on L-arginine-induced acute pancreatitis. Mice received intraperitoneal injections of either 8% L-arginine (pH 7.4, 2 × 4 g/kg, at 1 h interval) and were sacrificed at 72 h after the first L-arginine injection: **(A)** Effects of GPRC6A antagonist Cpd1 *in vivo*, representative images of pancreatic histopathology (H&E, ×200, scale bar 50 μm) and scores – **(B)** overall, **(C)** oedema, **(D)** inflammation, and **(E)** necrosis. Values are means ± SEM from six mice/group.

These data demonstrate that the different basic AAs have somewhat different effects on the isolated pancreatic acinar cells and that members of class C GPCRs CaSR and GPRC6A partially mediate cell responses. As opposed to the pharmacological profile of L-arginine effects *in vitro*, pancreatic histopathology and biochemical parameters of ARG-AP were unaltered by prophylactic administration of NPS-2143 or calindol (Figures 4C–G and Supplementary Figures S4A–C), or by continuous dosing of Cpd1 by implanted osmotic pumps (Figure 5). While NPS-2143 had no effect on ARG-AP associated elevation of lung MPO and serum IL-6 levels, Calindol significantly reduced these parameters (Supplementary Figures S4D,E).

As with caffeine (Figures 1A–F), such lack of protection by NPS-2143 and Cpd1 against ARG-AP was matched by *in vitro* profile of L-ornithine unresponsive to those drugs rather than of L-arginine.

### NPS-2143 Ameliorates Severity of CER-AP

**FIGURE 6:**
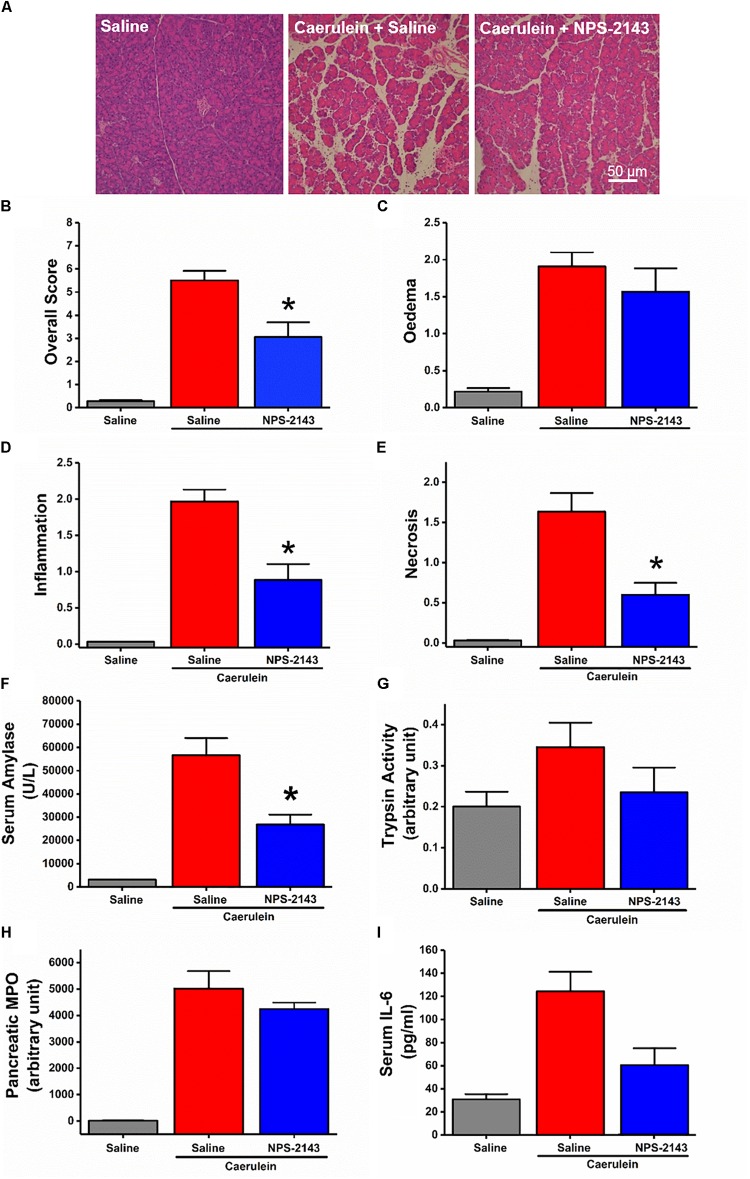
Effect of NPS-2143 on caerulein-induced acute pancreatitis. Mice received intraperitoneal injections of caerulein (7 × 50 μg/kg, at 1 h interval) and were sacrificed at 12 h after the first caerulein injection: **(A)** representative pancreatic histopathology (H&E, ×200, scale bar 50 μm) and scores – **(B)** overall, **(C)** oedema, **(D)** inflammation, and **(E)** necrosis; **(F)** serum amylase, **(G)** pancreatic trypsin activity, **(H)** pancreatic myeloperoxidase (MPO) activity, and **(I)** serum interleukin (IL-6) levels. *p* < 0.05 vs. caerulein treatment only. Values are means ± SEM from six mice/group.

Despite failure of NPS-2143 to protect mice against ARG-AP, we determined the effect of this compound in a standard model of hyperstimulation CER-AP. NPS-2143 improved pancreatic morphology (Figure 6A) and significantly reduced the overall histopathology score (Figure 6B), inflammation score (Figure 6D), necrosis score (Figure 6E) and serum amylase (Figure 6F). There was a tendency towards to reduced oedema score (Figure 6C), trypsin activity (Figure 6G), pancreatic myeloperoxidase activity (Figure 6H), as well as serum IL-6 levels (Figure 6I), but statistical significance was not reached.

## Discussion

Our data demonstrate that *in vitro* responses to various basic AAs have some important differences. The experiments with caffeine, which in pancreatic acinar cells blocks intracellular calcium signaling mediated by IP_3_ receptors (Huang et al., 2017), indicate involvement of calcium signaling in the *in vitro* responses to L-arginine. However, that does not seem to be relevant to ARG-AP. Surprisingly, L-histidine-induced cell death was exacerbated by caffeine *in vitro*, yet cyclophilin D knock-out showed protection against this AA both *in vitro* and *in vivo*. Altogether these data are consistent with the recent finding (Biczo et al., 2018) that cytosolic calcium was not central in AA-induced toxicity in the pancreas, although caffeine does not inhibit all components of calcium signaling or overload. In our hands, cyclophilin D knock-out could not ameliorate ARG-AP. While in their study (Biczo et al., 2018) the authors used the 3.3 × 3 g/kg regimen, we used a standard 2 × 4 g/kg protocol, as 3 × 3 g/kg regimen led to near 100% mortality in our mice. It has been described previously that rats injected with high doses of L-arginine fall into three groups of weak, normal, and strong responders (Bohus et al., 2008). We believe that such a distribution of responders may be differently affected in Ppif^-/-^ and Wt mice by unknown factors and eventually create differences in the results with respect to cyclophilin D knock-out. For instance, while doing our experiments, we noticed that the C57BL/6J mice that weighted less than 20 g were predominantly weak responders to L-arginine. Therefore, we decided to use only mice above 25 g in our work. One more factor that could underlie the lack of protection against L-arginine and L-ornithine in Ppif^-/-^ mice in our experiments was the reported ability of L-ornithine to produce reactive oxygen species in the acinar cells (Chvanov et al., 2015). The effects of oxidative stress on bioenergetics in these cells was later shown to be independent of cyclophilin D (Armstrong et al., 2018). The variability in results produced in AA based models of AP is not uncommon and may come down to the fact that the dose of AAs sufficient to trigger AP is very close to the dose of AAs that cause systemic toxicity and mortality, so that the disease is difficult to control (Kui et al., 2014). As a result the AA dose-dependence of disease severity is quite steep and for some of the parameters is bell-shaped (Tashiro et al., 2001), leading to over-sensitivity of the results to minor changes in parameters and protocols.

We found that *in vitro* necrotic cell death pathway activation of freshly isolated pancreatic acinar cells by L-arginine can be reduced by caffeine, a calcilytic NPS-2143, a calcimimetic calindol and a GPRC6A specific antagonist Cpd1. At the same time, none of these compounds could rescue pancreatic tissue from damage by L-arginine *in vivo*. This *in vivo* pattern can be matched by that of L-ornithine *in vitro*, where damage induced by L-ornithine is equally insensitive to any of these chemicals. The temporal metabolomics study in rats (Bohus et al., 2008) showed that during the first 8 h of ARG-AP there is substantial conversion of L-arginine to L-ornithine and urea. It has also been shown that inhibition of arginase, which converts L-arginine to L-ornithine, ameliorates ARG-AP in rats (Biczo et al., 2010). Thus our data support and provide further evidence that L-arginine to L-ornithine conversion, which is part of the urea cycle, is an important step in the induction of ARG-AP. It has also been documented previously that L-ornithine elicits mitochondrial depolarization and potentially toxic reactive oxygen species production in pancreatic acinar cells (Chvanov et al., 2015). Individuals who lack ornithine transcarbamylase (OTC), functional enzyme that converts L-ornithine into L-citrulline in the mitochondria, are at risk of AP (Anadiotis et al., 2001; Prada et al., 2012; Machado et al., 2013). It is also notable that the mitochondrial isoform of arginase (Arg2) plays a critical role in obesity-associated pancreatic cancer (Zaytouni et al., 2017). These data may signify specific importance of L-ornithine elevation in pancreatic mitochondria in a range of pancreatic pathologies.

It has been known for the last 60 years that non-natural or excessive doses of natural AAs, including ethionine, methionine, azaserine, cycloleucine, β-3-thienyl-DL-alanine, β-3-furyl-DL-alanine and, more recently, L-arginine, L-ornithine and L-lysine can cause lesions of the exocrine pancreas. Longnecker Longnecker (1977) stated that “the pancreas has been shown to concentrate both normal and several abnormal amino acids to a greater degree than most other tissues”. Such a propensity to concentrate AAs may be utilized in drug development by attaching an AA to an active small molecule drug destined for the pancreas. Thus, it is important to understand the mechanisms of AA toxicity, when it arises. Pancreatic acinar cells show the fastest protein synthesis among many cell types (Leblond et al., 1957). Mechanistically, the putative damage to protein synthesis by excess dietary or exogenous AAs was postulated to fall into three categories: imbalance, toxicity, and antagonism (Harper, 1956). Plasma AA imbalance in patients undergoing L-asparaginase chemotherapy (Minowa et al., 2012) may contribute to the high risk of AP associated with this treatment, in addition to the identified calcium overload (Peng et al., 2016). It is feasible that in these patients as well as in the animal models the damage to acinar protein synthesis plays a significant role in the initiation of the AP via an unfolded protein response and ER stress. An antibiotic puromycin, which blocks protein synthesis and generates ER stress, causes necrosis specifically in pancreatic acinar cells and only a few other cell types when injected intraperitoneally in rats (Longnecker and Farber, 1967; Longnecker et al., 1968). In relation to the AA experimental models of AP, ER stress was described as a starting event in ARG-AP in rats (Kubisch et al., 2006). By contrast, another recent comprehensive work identified mitochondrial injury (without prior calcium overload) as an initiator of damage (Biczo et al., 2018). The same mechanism was also suggested in choline deficient, ethionine-supplemented diet-induced AP.

The differences between the patterns of toxicity in the pancreas caused by different AAs have been known previously. For example, an ultrastructural study of pancreatic damage caused by L-arginine in rats highlighted the changes in the ER as preceding those in the mitochondria (Kishino and Kawamura, 1984). By contrast, upon injection of L-lysine in rats the same research group observed the reverse sequence of events (Kitajima and Kishino, 1985). In that respect, ethionine is closer to L-arginine than to L-lysine, because the ultrastructural changes in the ER after ethionine administration come earlier than those in mitochondria (Herman and Fitzgerald, 1962). While the damage caused by ethionine is augmented by a choline deficient diet, malnourishment of the experimental animals increases the pancreatic injury brought about by L-arginine (Takama and Kishino, 1985). However, whereas L- and D-ethionine are equally effective (Wachstein and Meisel, 1953), only the L-enantiomer of arginine has been shown to cause AP (Dawra et al., 2007). The fine differences between the effects of various AAs may indicate existence of individual AA-specific components of pancreatic toxicity in addition to a general one, which presumably unifies L-arginine, L-lysine, L-ornithine and ethionine and is mitochondria-linked (Biczo et al., 2018). Excessive histidine (Frezza, 2018), choline-deficient, ethionine-supplemented diet (Longnecker, 2002) and L-arginine (through NO synthesis) are known to reduce tetrahydrofolate availability. This would interfere with cytosolic and mitochondrial steps of one-carbon metabolism in the pancreas (Balaghi and Wagner, 1995; Horne and Holloway, 1997) and as such represents a shared pathway for these AAs. At the same time, it is feasible that some AA-specific pathways are related to protein synthesis (Weaver et al., 1994). Despite necroptosis having been shown to be an important mode of acinar cell death in AP models induced by CER (Wang et al., 2016) and bile acid (Louhimo et al., 2016), our findings rule out necroptosis as a significant contributor to the necrotic cell death caused by basic AAs.

We were successful in generating AP in mice injected with 2 × 4 g/kg L-histidine free base. Previous assessment of this AA for effects on rats was done with a lower dose of 1 × 3 g/kg L-histidine-HCl and reported no damage to the pancreas (Biczo et al., 2011a). That report highlighted the difficulty of working with L-histidine *in vivo* arising from its poor solubility, leading to introduction a lot of fluid into the animals. Indeed, we had to injection large volumes of freshly prepared 7% stock solution by microwaving and cooling to physiological temperatures. The model HIS-AP was different from ARG-AP as the severity of the disease caused by L-histidine was markedly decreased in Ppif^-/-^ mice. The cell death *in vitro* and *in vivo* observed in the presence of high levels of L-histidine adds to safety concerns of using histidine-tryptophan-ketoglutarate (HTK) solution for pancreas transplant preservation (Alonso et al., 2008; Troppmann, 2010). HTK solution is normally used cold, and we see no cell death *in vitro* in the presence of 20 mM L-histidine in our experiments if we drop the temperature from 37°C to room temperature (22–24°C, the data not shown). However, the very high concentration of histidine in HTK solution (300 mM) may still pose a risk for the transplants once the circulation is restored and body temperature reached.

The members of GPCR class C, CaSR, and GPRC6A, in addition to being sensors of the extracellular calcium level, also serve as nutrient (such as L-AA and polyamine) sensors. The former receptor responds to aromatic L-AAs phenylalanine, tryptophan and histidine with EC_50_ at low micromolar concentrations, while the latter receptor senses L-arginine and L-lysine (Conigrave and Hampson, 2006; Wellendorph et al., 2009). CaSR is expressed in human pancreatic exocrine (including acinar) and endocrine cells (Racz et al., 2002). In rats around 50% of pancreatic acinar cells show functional responses to CaSR stimulation by inorganic cations Gd^3+^ or 8 mM extracellular Ca^2+^, however, pancreatic acinar cells do not respond to millimolar concentrations of an organic polycation neomycin known to activate CaSR elsewhere (Bruce et al., 1999). It has also been reported that intravenous AAs, which should in theory activate CaSR and GPRC6a, do not stimulate exocrine pancreatic secretion (Stabile et al., 1984). Using RT-PCR, GPRC6A expression has been detected in the pancreas, although the main signal may come from endocrine cells (Wellendorph and Brauner-Osborne, 2004). We confirmed the presence of CaSR and GPRC6A receptors in murine and human pancreatic acinar cells by immunocytochemistry. We investigated whether these receptors play any role in ARG-AP but reached a negative conclusion, based on no effect of NPS-2143 (CaSR and to less extent GPRC6A inhibitor), calindol (CaSR activator), or Cpd1 (GPRC6A inhibitor). The *in vitro* cell death caused by L-arginine was, however, dampened by NPS-2143, Cpd1 calindol, and R-568. The *in vitro* responses to L-histidine were inhibited by Cpd1, while responses to L-ornithine were insensitive to any of these GPCR modulators.

In contrast to ARG-AP, NPS-2143 administration substantially ameliorated the severity of CER-AP. This is an encouraging finding, because it may have some therapeutic promise for the human disease. From one point of view, it is known that AP is associated with hyperparathyroidism (and hence hypercalcaemia) (Biondi et al., 2011) or post-operative hypercalcaemia following liver transplantation (Chung et al., 2011). On the other hand, severe AP is associated with “true” hypocalcaemia and low levels of parathyroid hormone (McMahon et al., 1978). NPS-2143 has a long half-life in the circulation (Gowen et al., 2000) and may therefore represent a lead compound as a treatment option to restore systemic calcium post-induction of AP. NPS-2143 is an R-enantiomer belonging to the biaryloxypropanol class of molecules. Calcilytics NPS-2143, encaleret, and ronacaleret are closely structurally related to several third generation adrenergic antagonists, such as (in order of decreasing similarity) bucindolol, carvedilol, naftopidil, and nebivolol. Carvedilol (a non-selective β blocker/α1 blocker), nebivolol (β1 receptor blocker), naftopidil (α1 blocker), and a closely linked non-AR drug ranolazine are used clinically as racemates of R- and S-enantiomers. Whereas anti-β-adrenergic activity of NPS-2143 and other biaryloxypropanol calcilytics is recognized (notably for the S-enantiomers) (Marquis et al., 2009; Widler, 2011), the interaction between the third generation beta antagonists and CaSR/GPRC6A has never been a subject of a scrutiny. However SAR studies suggest that transformations from NPS-2143 to S-bucindolol and S-carvedilol should generally retain calcilytic activity (Yang et al., 2005; Balan et al., 2009; Shinagawa et al., 2010; Kiefer et al., 2011; Shinagawa et al., 2011). Indeed, the reported IC50 for the CaSR of the Cpd9 variant of NPS-2143 (bucindolol) is 100 nm, not that different from NPS-2143 itself at 50 nm (Gavai et al., 2005). Since carvedilol and nebivolol are prescribed and deemed to be clinically safe (despite some anti-hERG activity), they may offer an alternative to NPS-2143 and may be worthy of further investigation in AP.

Our data provide evidence for the role of L-arginine to L-ornithine conversion in the pathogenesis of ARG-AP. The role of systemic factors in this conversion, which is part of the urea cycle and ammonia detoxification, requires further elucidation. Our results also point to little role of intracellular calcium signaling or surface GPCR class C in ARG-AP. The fine differences between the pharmacological profiles of the effects of various basic AAs suggest additional non-mitochondrial mechanisms of pancreatic toxicity that may occur via disruption of protein synthesis or ER stress.

## Author Contributions

XZ, TJ, NS, WH, and MC performed the *in vitro* and *in vivo* experiments, analyzed and interpreted the data. LY, XY, CH, and DD involved in molecular biology experiments. LW participated in caerulein-induced acute pancreatitis experiment in mice. PS, TL, QX, and DC supervised the students and involved in data interpretation. RM provided the Ppif^-/-^ mice and involved in data interpretation. WH, MC, and RS obtained the funding, designed the study, supervised the students, drafted and critically revised the manuscript.

## Conflict of Interest Statement

The authors declare that the research was conducted in the absence of any commercial or financial relationships that could be construed as a potential conflict of interest.

## Supplementary Material

Figure S1 The procedure of calculating time to half-maximal response (HMR) of necrotic cell death *in vitro*. **(A)** Traces of propidium iodide (PI) fluorescence uptake from the individual experiments of cells treated with L-arginine that correspond to Figure 1Fi. **(B)** The mean ± SEM for L-arginine and L-arginine plus caffeine (2 and 5 mM) treatment groups corresponding to Figure 1Fi as well as a control group; the black trace in the figure **(B)** is obtained by averaging the individual experiments from **(A)**. **(C)** The calculation of the time to HMR for the black trace from **(A)**. **(D)** Is identical to Figure 1Fi and is obtained by averaging all data from treatment groups on **(B)** according to the procedure from **(C)**. ^∗^*p* < 0.05 vs. L-arginine treatment only. Values are means ± SEM from ≥6 experiments/group.

Figure S2 Effects of necroptosis modulator on pancreatic cell death caused by basic amino acids *in vitro*. Necroptosis modulators: 50 μM of RIPK1 inhibitor necrostatin-1 (Nec-1), 25 μM of pan-caspase inhibitor z-VAD-FMK (z-VAD), 3 μM of RIPK3 inhibitor GSK-872, and 0.5 μM of MLKL inhibitor necrosulfonamide (NSA). Necrotic cell death pathway activation (presented as time to half-maximal response [HMR] of propidium iodide [PI] uptake) of freshly isolated mouse pancreatic acinar cells caused by basic amino acids: **(A)** L-arginine, **(B)** L-ornithine, and **(C)** L-histidine. ^∗^*p* < 0.05 vs. corresponding L-AA treatment only. Values are means ± SEM from ≥6 experiments/group.

Figure S3 Representative histopathological images for experimental groups in the L-arginine- and L-histidine-induced acute pancreatitis. Cyclophilin D knock-out (Ppif^-/-^) or wild type (Wt) mice received either intraperitoneal injections of either 8% L-arginine or 7% L-histidine (both: pH 7.4, 2 × 4 g/kg, at 1 h intervals) and were sacrificed at 72 h after the first L-arginine/L-histidine injection. Representative pancreatic histopathology for all groups (H&E, ×200, scale bar 50 μm) are shown.

Figure S4 Effect of allosteric modulators of GPCR Class C on biochemical markers of L-arginine-induced acute pancreatitis *in vivo*. Mice received intraperitoneal injections of either 8% L-arginine (pH 7.4, 2 × 4 g/kg, at 1 h interval) and were sacrificed at 72 h after the first L-arginine injection: **(A)** serum amylase, **(B)** pancreatic trypsin activity, **(C)** pancreatic myeloperoxidase (MPO) activity, **(D)** lung MPO activity, and **(E)** serum interleukin (IL)-6 levels. ^∗^*p* < 0.05 vs. L-arginine only. Values are means ± SEM from ≥6 mice/group.
