# Supplementary material for: Mechanisms of Pancreatic Injury Induced by Basic Amino Acids Differ Between L-Arginine, L-Ornithine, and L-Histidine
Source: Front Physiol. 2019 Jan 15;9:1922. doi: 10.3389/fphys.2018.01922 (PMC6341295; doi:10.3389/fphys.2018.01922)
Supplement: Figure S1 — The procedure of calculating time to half-maximal response (HMR) of necrotic cell death in vitro. (A) Traces of propidium iodide (PI) fluorescence uptake from the individual experiments of cells treated with L-arginine that correspond to Figure 1Fi. (B) The mean ± SEM for L-arginine and L-arginine plus caffeine (2 and 5 mM) treatment groups corresponding to Figure 1Fi as well as a control group; the black trace in the figure (B) is obtained by averaging the individual experiments from (A). (C) The calculation of the time to HMR for the black trace from (A). (D) Is identical to Figure 1Fi and is obtained by averaging all data from treatment groups on (B) according to the procedure from (C). ∗p < 0.05 vs. L-arginine treatment only. Values are means ± SEM from ≥6 experiments/group. [file Presentation_1.PPTX]

## Slide 1
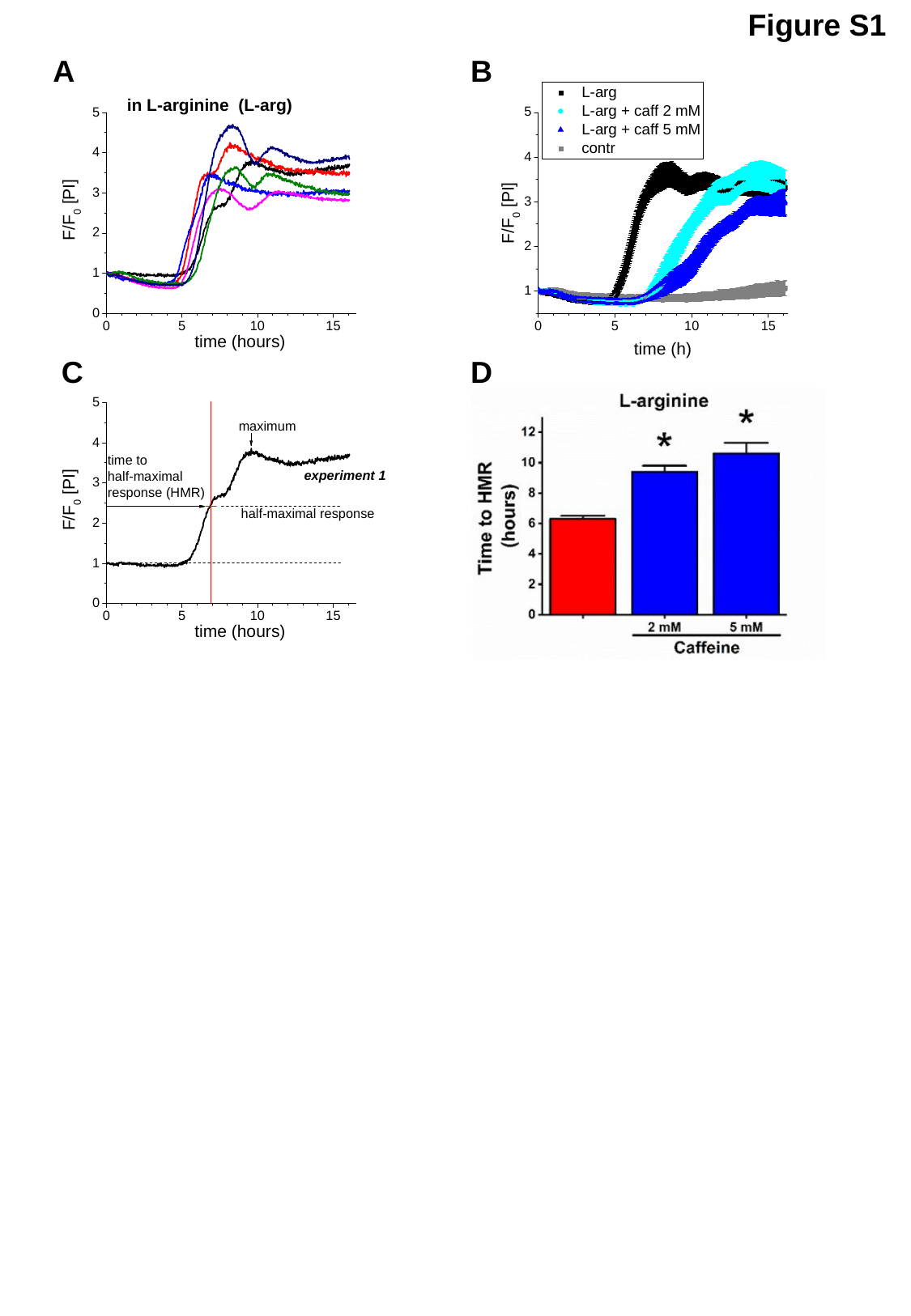

Figure S1
A
B
 C
D

## Slide 2
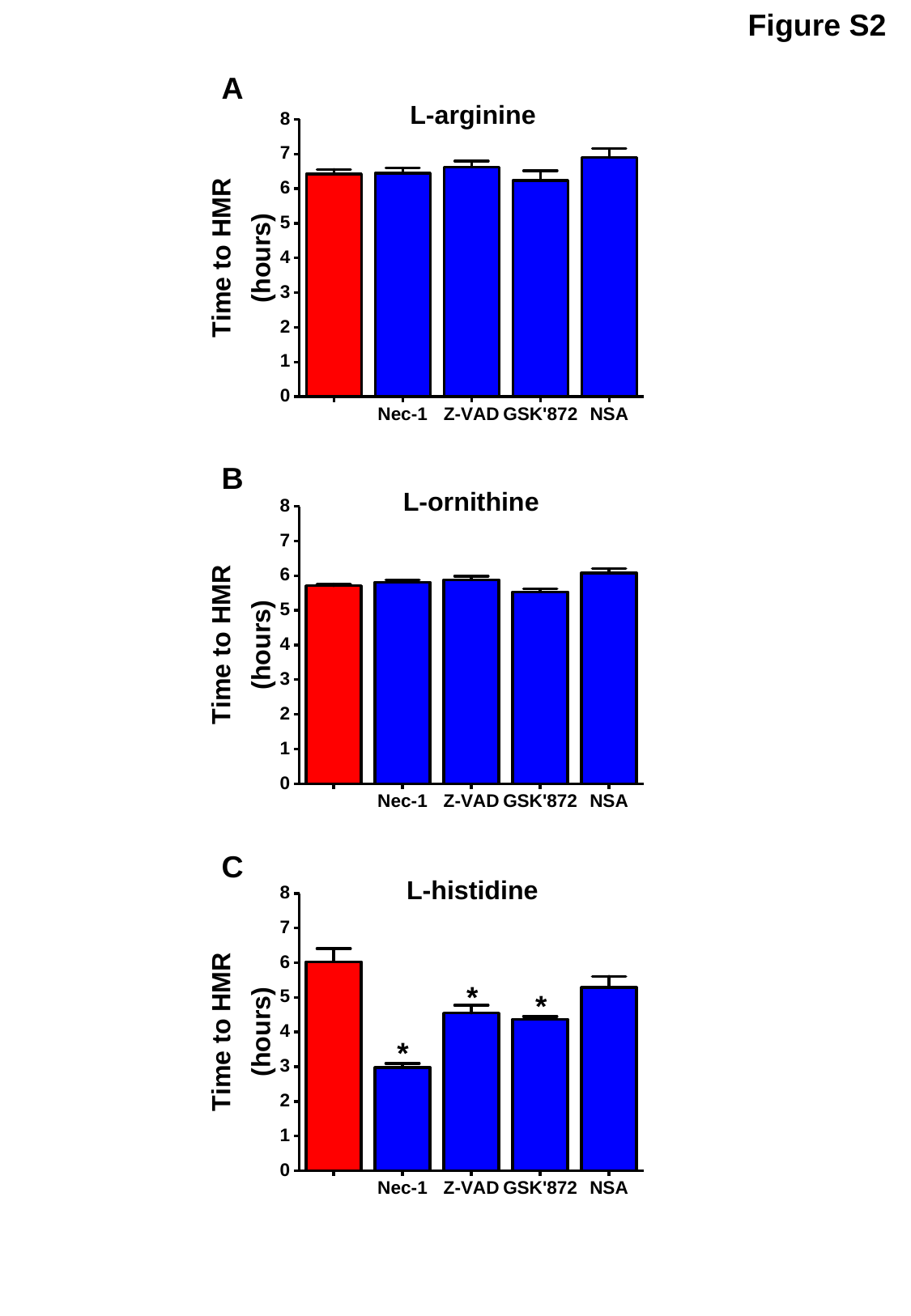

Figure S2
A
B
C

## Slide 3
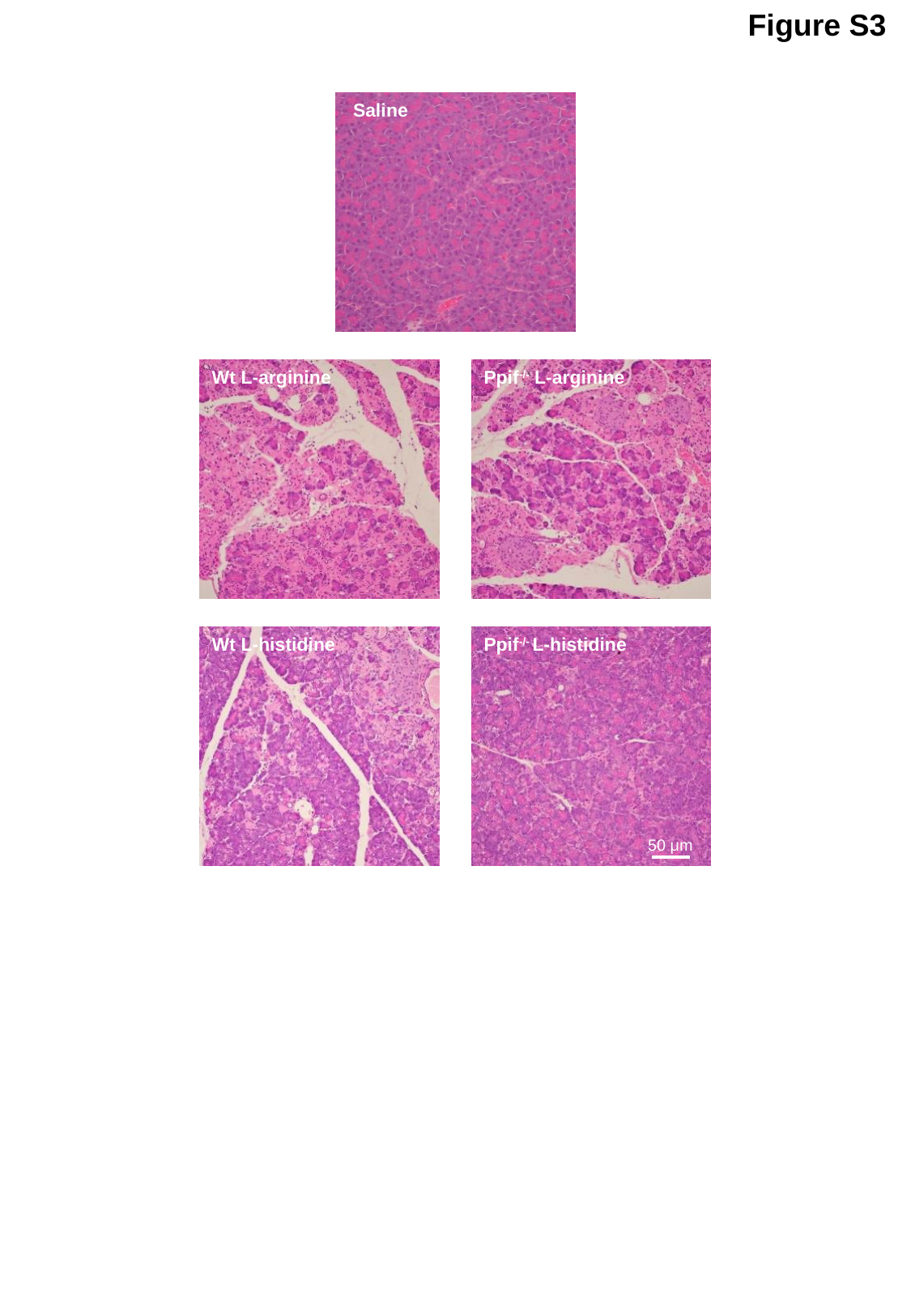

Figure S3
Wt L-arginine
Ppif-/- L-arginine
 Saline
Wt L-histidine
Ppif-/- L-histidine
50 μm

## Slide 4
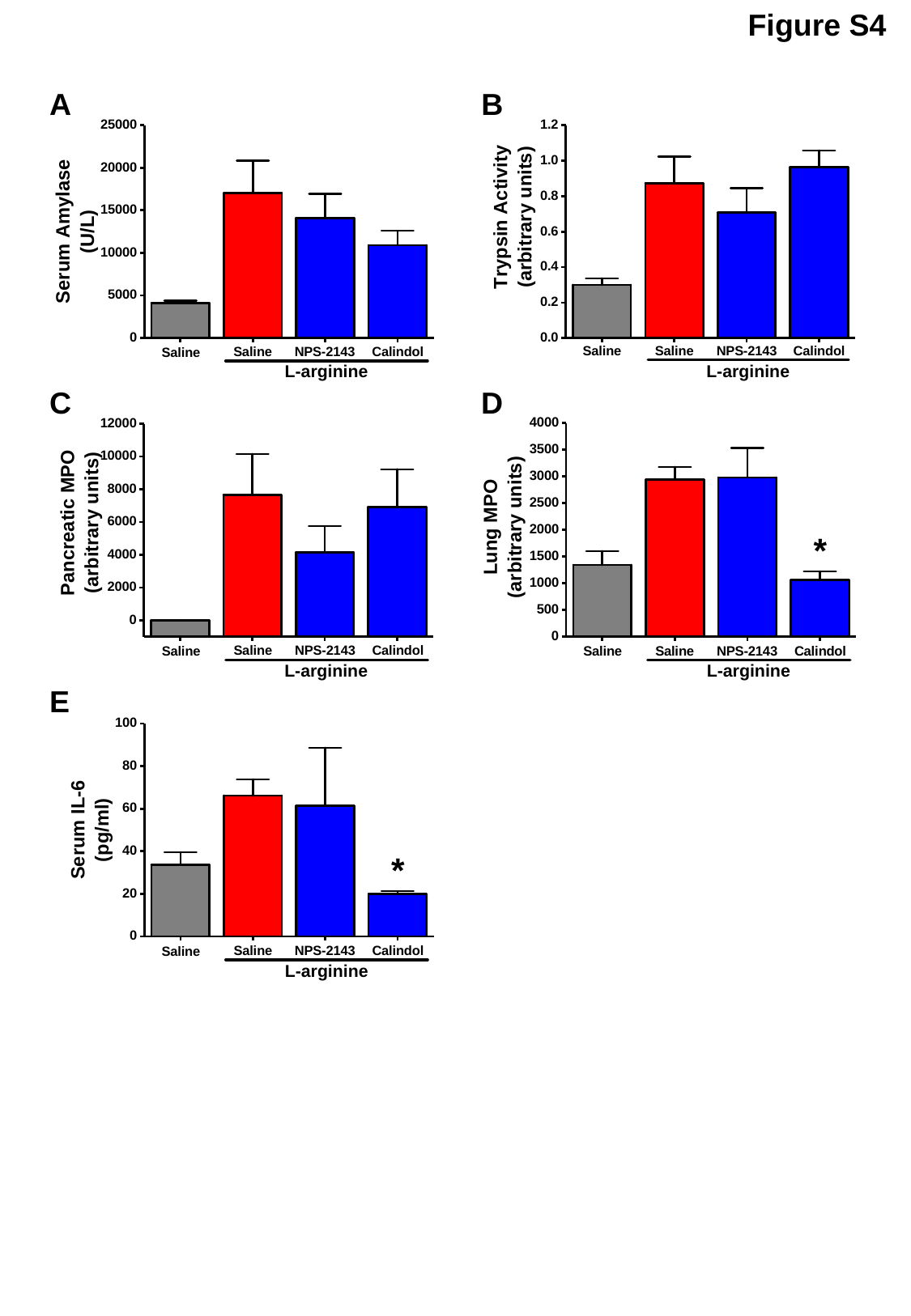

Figure S4
A
B
C
D
E
